# A case of solitary rectal diverticulum presenting with a large retrorectal abscess

**DOI:** 10.1016/j.amsu.2019.11.015

**Published:** 2019-11-27

**Authors:** Stefanos Gorgoraptis, Sofia Xenaki, Elias Athanasakis, Anna Daskalaki, Konstantinos Lasithiotakis, Evangelia Chrysou, Emmanuel Chrysos

**Affiliations:** a Department of General Surgery, University Hospital of Heraklion Crete, Greece; b Department of Radiology, University Hospital of Heraklion Crete, Greece

**Keywords:** Rectal diverticulum, Abscess, Diverticulitis, Complications

## Abstract

Colonic diverticular disease is a common condition, affecting 50% of the population aged above 80. In contrast, rectal diverticular disease is a rare condition with very few cases reported, while symptomatic rectal diverticular disease is even rarer. We present a case of a symptomatic large rectal diverticulum presenting with a retrorectal abscess. A 49-year-old Caucasian female was brought to the emergency department complaining of abdominal pain and weakness in the lower limbs. She was found to have obstructive uropathy and unilateral sciatic neuropathy. She rapidly developed acute abdomen and emergency laparotomy revealed a giant purulent rectal diverticulum. The patient underwent exploratory laparotomy and a loop colostomy was made to decompress the colon.

## Highlights

• Colonic diverticular disease is a common condition, affecting 50% of the population aged above 80.
• In contrast, rectal diverticular disease is a rare condition with very few cases reported, while symptomatic rectal diverticular disease is even rarer.

## 1 Introduction

Despite the high incidence of colonic diverticular disease, the occurrence of rectal diverticula is extremely unusual, with only few, sporadic published reports since 1911 [11]. According to the current literature, rectal diverticulosis applies for 0.1% of the cases of colonic diverticulosis. The vast majority of patients with rectal diverticula are diagnosed incidentally; symptomatic rectal diverticula are very rare [1,2]. We report an isolated rectal diverticulum, which presented with a large retrorectal abscess. The SCARE Guidelines list was followed [12].

## 2 Case presentation

A 49-year-old Caucasian female was brought to the emergency department complaining of abdominal pain and weakness in the lower limbs. She had developed lower abdominal pain radiating to the lumbar region, along with gradually deteriorating paraparesis 24 hours prior to her presentation. The patient's past medical history included a colectomy 17 years previously, due to endometriosis.

**Fig. 1:**
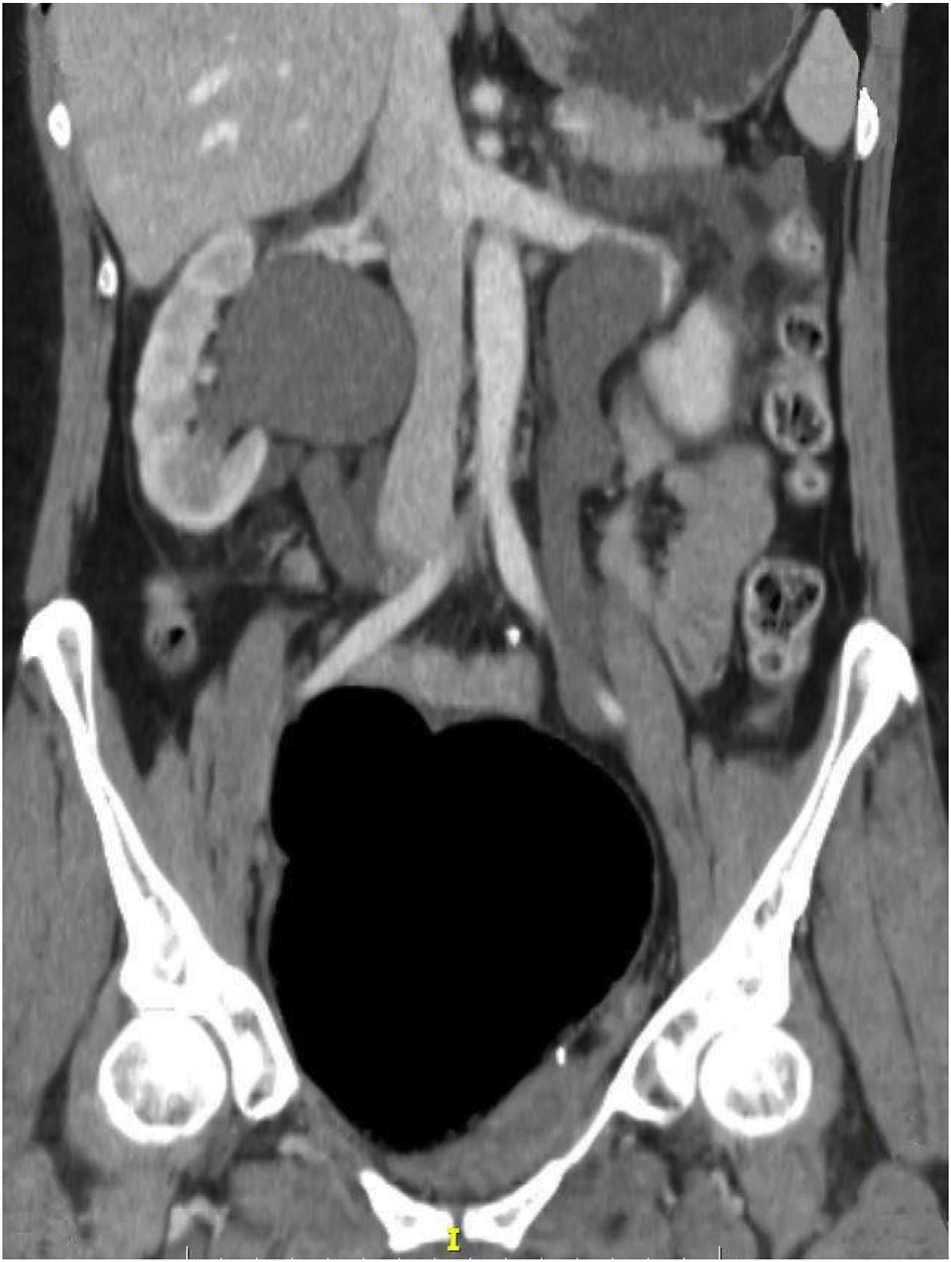
Axial CT image depicting obstructive uropathy due to gas-filled pelvic structure.

In the emergency department, the patient was anuric and tachycardic. Physical examination revealed diffuse abdominal tenderness on deep palpation and mild rebound (rigidity/tenderness). Digital rectal examination revealed stenosis of the colectomy's anastomosis. Neurologic examination revealed asymmetric paraparesis and hypoesthesia in the lower limbs, affecting hip extension, knee flexion, ankle dorsiflexion, plantarflexion, eversion and big toe extension, with brisk tendon reflexes in the knees but absent in the ankle, in keeping with bilateral sciatic neuropathy, worse on the left. Laboratory tests revealed leukocytosis and impaired renal function [WBC = 37.7 K/μl, 94.3% neutrophils, creatinine = 2.8 mg/dl]. Ultrasound and CT scan of the abdomen and pelvis was performed, which demonstrated a gas filled cavity rectal dilatation, pyeloureteral dilatation, in addition to free fluid and stranding of the perirenal area bilaterally, in keeping with obstructive uropathy, attributed to bilateral compression of the ureters by the gas filled cavity initially presumed to be dilated rectum (Fig. 1).

Bilateral nephrostomies were inserted. In the subsequent 24 hours the patient deteriorated with acute abdomen, bilateral sciatic neuropathy and sepsis. MRI and subsequent CT scan of the pelvis were performed, both of which demonstrated a sizeable (13 × 8 × 8 cm), thick-walled, enhancing, true rectal diverticulum, originating from the right lateral rectal wall, accompanied by extensive inflammation of pelvic structures (Fig. 2A, Fig. 2B, Fig. 3). Part of the small bowel was also found attached to the diverticulum, resulting to inflammation and ileus.

**Fig. 2A:**
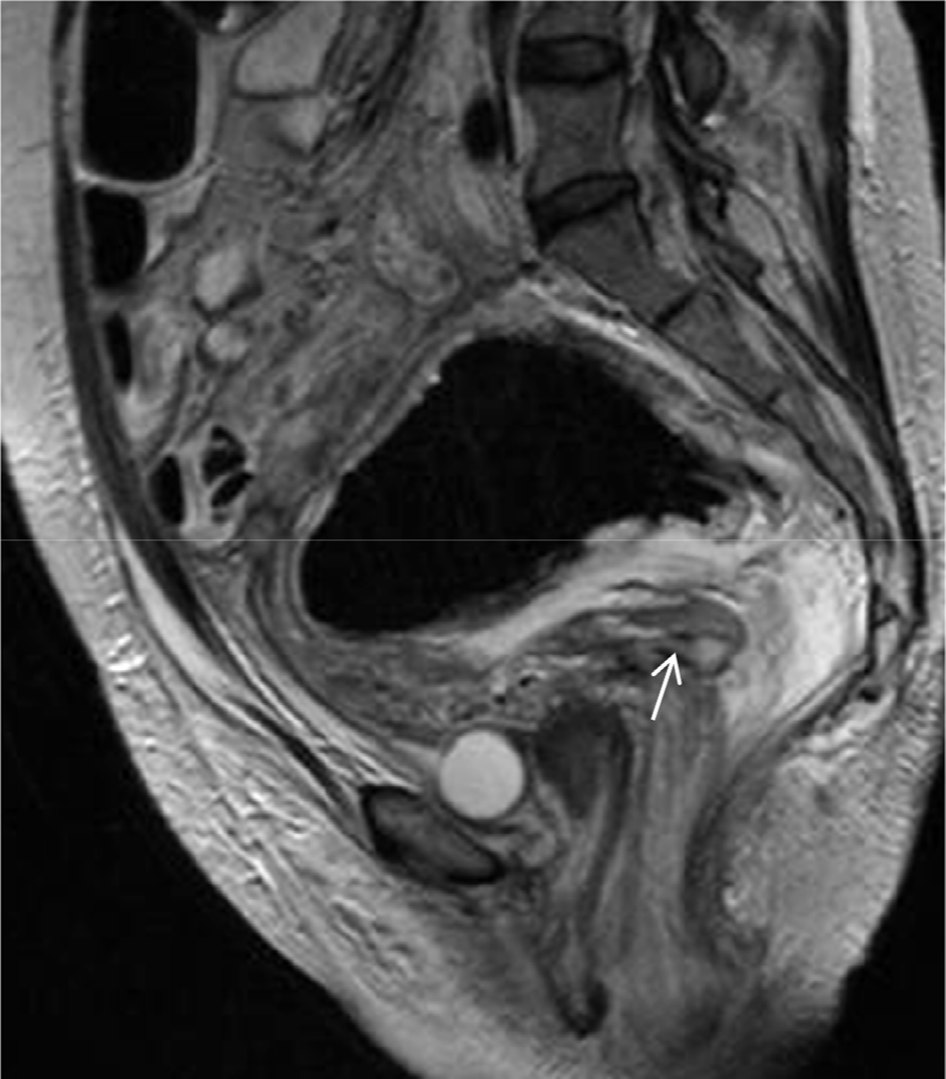
T2-weighted sagittal image: A thick –walled, flask-shaped structure predominately filled with air adjacent to the rectal wall. Arrow shows horizontal low signal intensity line corresponding to the diverticular sinus tract, as confirmed by subsequent CT.

**Fig. 2B:**
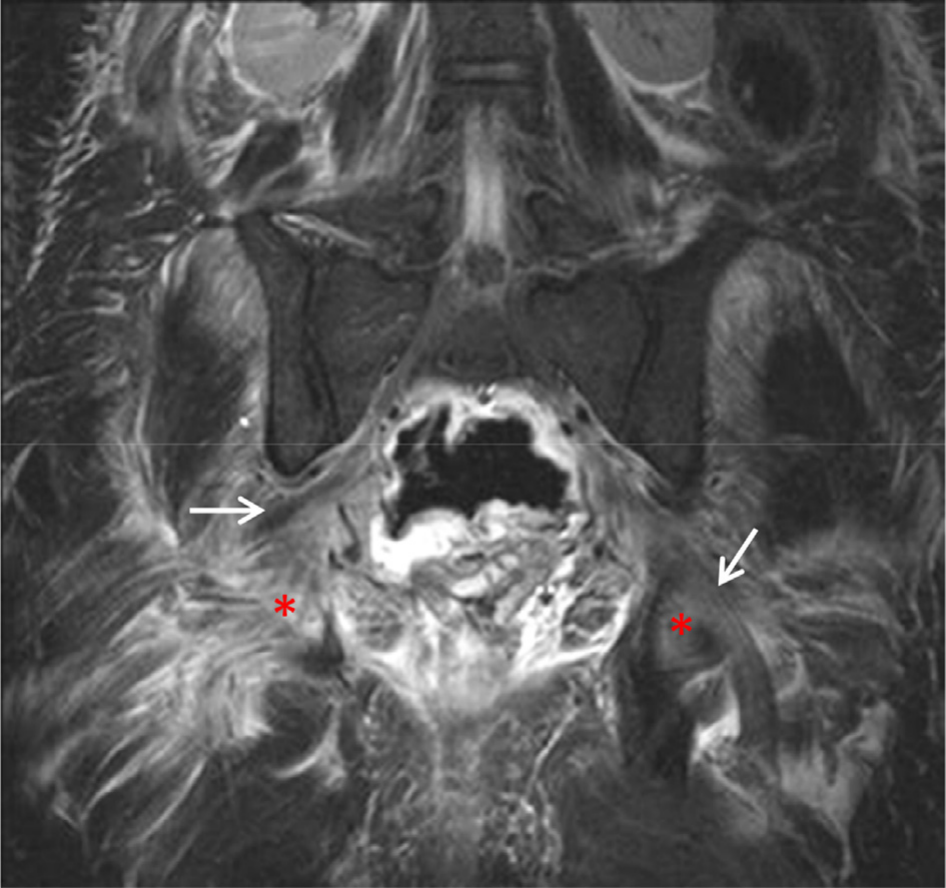
STIR coronal image shows diffuse, hyperintense sciatic nerve thickening (arrows), accompanied by edematous appearances of piriformis muscles (*), findings consistent with sciatic neuritis, due to compression by the inflamed rectal diverticulum.

**Fig. 3:**
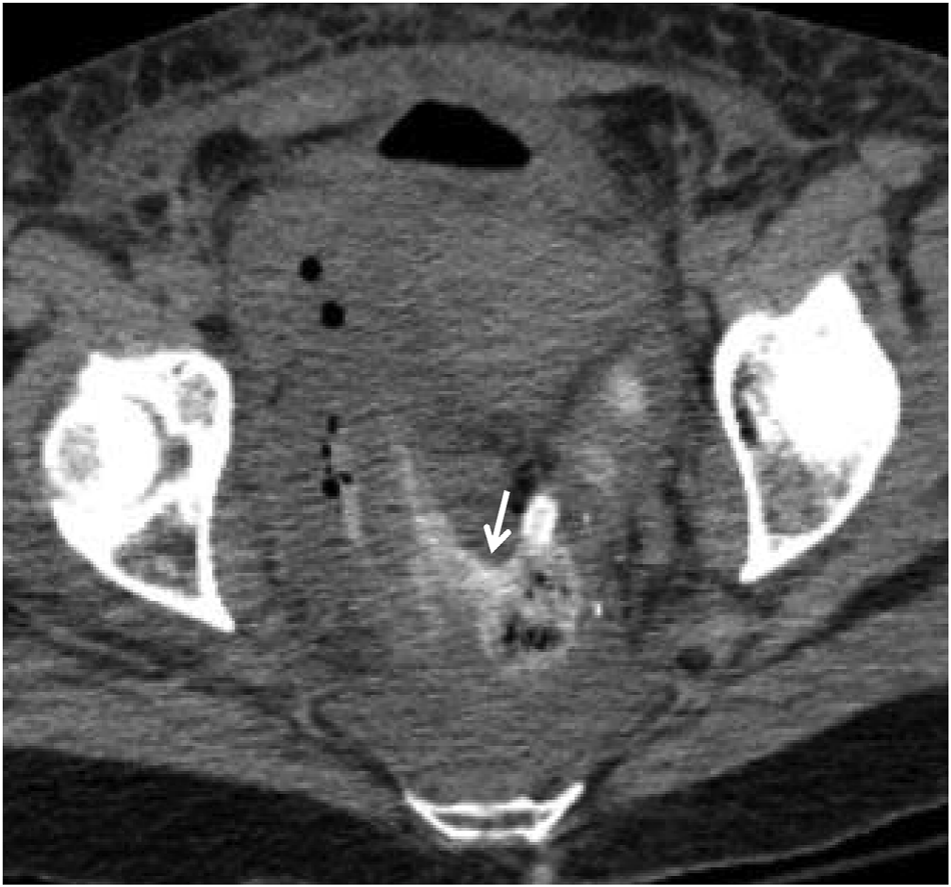
CT axial image: Retrograde filled rectum with water soluble contrast enema. A predominately gas-fluid cavity is shown in the pelvis adjacent to the right rectal wall, communicating with the rectal lumen, consistent with a giant rectal diverticulum (arrow depicts the neck of the diverticulum, connecting rectum with the diverticular cavity).

**Fig. 4:**
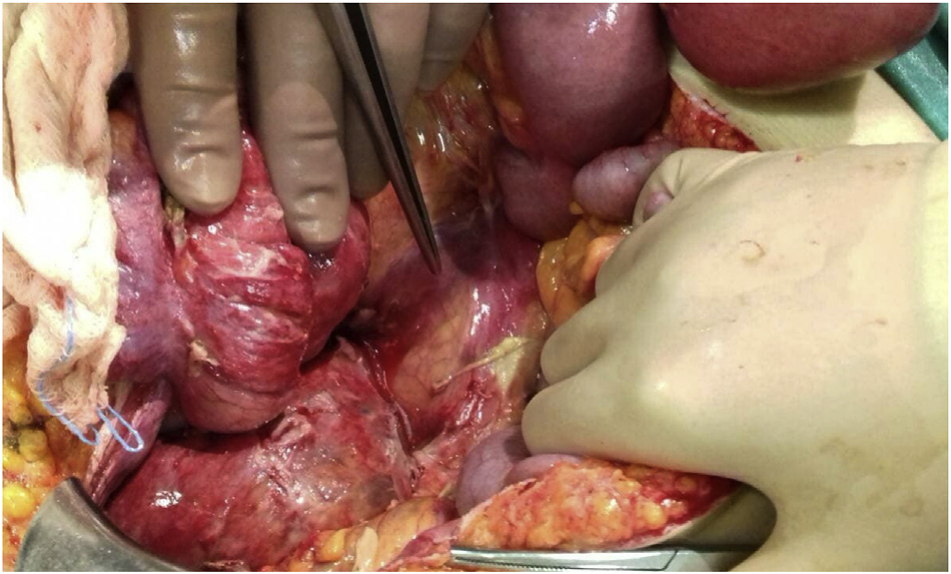
The diverticulum during operation.

The patient underwent exploratory laparotomy. A loop colostomy in the descending colon was made, in order to confront the acute abdomen and to decompress the obstruction of the upper rectum. The diverticulum was detected, strongly attached to the postperitoneum (Fig. 4). A peritoneal lavage was performed and a soft discharge was placed deep in the pelvis. We decided neither to excise nor to drain the diverticulum due to its spoilt and fragile walls. The patient stayed in the ICU until the third post-operative day. She had an uncomplicated postoperative period although her neurologic symptoms were present and mildly deteriorated on the fifth post-operative day. The patient elected to self-discharge and to continue her treatment in France, her country of origin. Unfortunately, she was lost to follow up.

## 3 Discussion

Diverticular disease of the colon is a common condition developed countries, affecting 50% of the population aged above 80. Colonic diverticula are acquired herniations of the mucosa and part of the submucosa through the muscularis propria. Diverticula are more frequently seen in the distal colon, with the sigmoid colon being affected in 90% of the patients [[1], [2], [3]].

In contrast, rectal diverticular disease is a rare condition with very few cases reported. They make up solely for 0.1% of colonic diverticular disease [1,2]. Two major theories about the low incidence of rectal diverticulosis have been reported. Firstly, the rectal wall is composed of three layers; muscularis externa, submucosa and mucosa. The muscularis externa is formed of two layers of smooth-muscle; longitudinal (superficial) and circular (deep). The longitudinal layer consists of the coalescence of the three colic taeniae; mesocolic, mesenteric and libera, forming a uniform circumferential supporting line. The circular fibers also form a continuous sheet along the entire length of the rectum. The combination of the longitudinal and the circular fibers forms a highly resistant wall. Secondly, less constant internal pressure is exerted on the rectum by accumulated feces and by a lower peristaltic activity as compared with the sigmoid colon [4].

In contrast to colonic diverticula, most rectal diverticula are true diverticula, involving all three layers of the rectal wall. In addition, they largely accompany colonic diverticulosis. Nevertheless, rectal pseudodiverticula and solitary rectal diverticula have been reported [5,8]. The most frequent number of rectal diverticula per patient is 1–3, with a mean diameter >2cm, larger than the rest of the colon [3]. According to Steenvoorde and colleagues, diverticula measuring 4 cm or more should be classified as giant diverticula [10]. Furthermore, the clinician should bear in mind that rectal diverticula change in diameter, depending on the intraabdominal pressure [1]. Rectal diverticula are usually located along the lateral walls of the rectum, compared with anterior or posterior locations. The taenia libera and the taenia omentalis merge over the anterior rectal wall and the taenia mesocolica covers the posterior rectal wall. As a result, rectum's circumferential supporting line tends to be thicker and stronger on the anteroposterior axis [1,9].

Two main factors contribute to the pathogenesis of rectal diverticula; 1. A pressure gradient between the bowel lumen and the mucosa; 2. Areas of relative weakness of the bowel wall. This theory is supported by the fact that rectal diverticula are true [1]. The predisposing factors that affect the development of rectal diverticula are congenital and acquired, coalescing to the formation of areas of focal weakness in the rectal wall. Congenital factors include weakness of the circular muscle layer, primary muscle atrophy, absence of support structures such as the coccyx. Acquired factors include relaxation of the rectovaginal septum, recurrent faecal impaction with increased intrarectal pressure and rectal distention, pelvic trauma, infections, obesity [3,4,7]. Surgical interventions have also become a major causative factor for rectal diverticula. Several authors have shown that surgical suture points on the rectal wall lead to focal weakness and predispose to the development of diverticula [1,6]. Resection of the distal rectum, in operations like the STARR or the Longo techniques, is total and extends to the circular musculature, thus forming a fibrous ring which tends to loosen with time. In our case, focal weakness at the point of colectomy's anastomosis is a likely cause for the formation of the rectal diverticulum.

Rectal diverticulosis is usually an incidental diagnosis. In some rare cases, rectal diverticula become symptomatic, presenting with symptoms of diverticulitis (pain, hematochezia, abscess formation, perforation), as well as strictures, ileus, change in bowel habits, rectovesical fistula, rectal prolapse, perineal mass and retrorectal cyst. To our knowledge, sciatic neuropathy and bilateral obstructive uropathy, as in our case, have never been reported before. Asymptomatic patients do not require treatment, whereas symptomatic rectal diverticulosis necessitates surgical intervention, if possible [2,3,7,8]. Acute excision or drainage of the diverticulum was not possible in our case, since the diverticular walls were spoilt, thus making the definitive surgical treatment of the diverticulum extremely dangerous.

In conclusion, clinicians should be aware of symptomatic rectal diverticulosis as a rare but potentially clinically significant complication of rectal surgery. Operations in the rectal wall which affect either the continuity or the muscular lining, may form points of least resistance, thus predisposing for the development of rectal diverticula. In our case, it emerged clinically as a large retrorectal abscess, requiring prompt intervention.

## Ethical approval

Authors are aware that Annals of Med and Surg is committed to following the highest standards of publication ethics as promoted by the Committee on.

Publication Ethics.

## Sources of funding

No funding.

## Author contribution

Stefanos Gorgoraptis, Medical Student: contribution in the narration of the manuscript.

Sofia Xenaki, MD MSc FACS mESCP (Equally contributed): contribution in the narration of the manuscript, assisted in the operation and contributed in the collection of data.

Elias Athanasakis, MD PhD: Surgeon and contribution in the narration of the manuscript.

Anna Daskalaki, MD: contribution in the narration of the manuscript.

Konstantinos Lasithiotakis, MD PhD FEBS: reviewed the manuscript.

Evangelia Chrysou, MD PhD: was the specialized Colorectal Radiologist.

Emmanuel Chrysos, MD PhD FACS: was the Prof of the Department and reviewed the manuscript.

## Trial registry number

4980.

## Guarantor

Emmanuel Chrysos MD PhD FACS.

Professor of Surgery.

Head of the Surgical Department.

## Consent

The patient has given consent for possible publication of this case report.

## Patient's consent

The patient has given consent for possible publication of this case report.

## Provenance and peer review

Not commissioned, externally peer reviewed.

## Declaration of competing interest

The Authors declare that have no Conflict of Interest.
